# Purpose in Life Predicts Better Emotional Recovery from Negative Stimuli

**DOI:** 10.1371/journal.pone.0080329

**Published:** 2013-11-13

**Authors:** Stacey M. Schaefer, Jennifer Morozink Boylan, Carien M. van Reekum, Regina C. Lapate, Catherine J. Norris, Carol D. Ryff, Richard J. Davidson

**Affiliations:** 1 Department of Psychology, University of Wisconsin – Madison, Madison, Wisconsin, United States of America; 2 Waisman Laboratory for Brain Imaging and Behavior, University of Wisconsin – Madison, Madison, Wisconsin, United States of America; 3 Center for Investigating Healthy Minds, University of Wisconsin – Madison, Madison, Wisconsin, United States of America; 4 Center for Women's Health and Health Disparities Research, University of Wisconsin – Madison, Madison, Wisconsin, United States of America; 5 Institute on Aging, University of Wisconsin – Madison, Madison, Wisconsin, United States of America; 6 Centre for Integrative Neuroscience and Neurodynamics, School of Psychology and Clinical Language Sciences, University of Reading, Reading, United Kingdom; 7 Department of Psychology, Swarthmore College, Swarthmore, Pennsylvania, United States of America; University of Leicester, United Kingdom

## Abstract

Purpose in life predicts both health and longevity suggesting that the ability to find meaning from life’s experiences, especially when confronting life’s challenges, may be a mechanism underlying resilience. Having purpose in life may motivate reframing stressful situations to deal with them more productively, thereby facilitating recovery from stress and trauma. In turn, enhanced ability to recover from negative events may allow a person to achieve or maintain a feeling of greater purpose in life over time. In a large sample of adults (aged 36-84 years) from the MIDUS study (Midlife in the U.S., http://www.midus.wisc.edu/), we tested whether purpose in life was associated with better emotional recovery following exposure to negative picture stimuli indexed by the magnitude of the eyeblink startle reflex (EBR), a measure sensitive to emotional state. We differentiated between initial emotional reactivity (during stimulus presentation) and emotional recovery (occurring after stimulus offset). Greater purpose in life, assessed over two years prior, predicted better recovery from negative stimuli indexed by a smaller eyeblink after negative pictures offset, even after controlling for initial reactivity to the stimuli during the picture presentation, gender, age, trait affect, and other well-being dimensions. These data suggest a proximal mechanism by which purpose in life may afford protection from negative events and confer resilience is through enhanced automatic emotion regulation after negative emotional provocation.

## Introduction

Growing evidence from epidemiological research suggests that self-reported psychological well-being is important for both health and longevity, potentially through mechanisms promoting resilience in the face of adversity (see [1], [2] for recent theoretical reviews). Ryff defined psychological well-being in terms of six key dimensions: autonomy (capacity for self-determination), environmental mastery (ability to manage one’s surrounding world), personal growth (realization of potential), positive relations with others (high-quality relationships), purpose in life (meaning and direction in life), and self-acceptance (positive self-regard) [3], [4]. Higher levels of purpose in life, personal growth, and positive relations have been linked to lower cardiovascular risk (lower glycosylated hemoglobin, lower weight, lower waist-hip ratios, and higher “good” cholesterol (high-density lipoprotein (HDL)) as well as better neuroendocrine regulation (lower salivary cortisol throughout the day) [5]. Higher profiles on purpose in life and positive relations with others have also been linked to lower inflammatory factors: interleukin-6 (IL-6) and its soluble receptor (sIL-64) [6], providing empirical support linking these well-being dimensions to better health profiles.

Recent evidence suggests that relative to other dimensions of well-being, purpose in life appears to be particularly important in predicting future health and mortality. In a prospective, longitudinal, epidemiological study of community-dwelling older persons without dementia (Rush Memory and Aging Project), greater purpose in life was associated with better ability to perform day-to-day activities and less mobility disability in the future [7]. Those who reported greater purpose in life exhibited better cognition at follow-up, had a reduced risk of mild cognitive impairment, and a slower rate of cognitive decline [8]. In fact, people who reported high levels of purpose in life (90^th^ percentile or higher) were 2.4 times more likely to remain free of Alzheimer Disease than people who reported low levels (10^th^ percentile or lower). Moreover, on postmortem examination of the brain for Alzheimer Disease-related pathology, purpose in life modified the associations between cognition and both global pathologic change and plaque accumulation [9], suggesting that having greater purpose in life may protect against the detrimental effects of aging-related changes in the brain that have been linked to Alzheimer Disease. Finally, greater purpose in life was associated with a reduced risk of mortality from all causes [10]. Collectively, these findings suggest that the ability to find meaning and direction in life may help buffer or slow the effects of aging and even the ultimate outcome: death.

Besides healthier biomarker levels, slowed effects of aging, and increased longevity, higher levels of psychological well-being have also been associated with lower rates of depression [3], [4], [11], with the dimension of purpose in life consistently showing negative relations with depressive symptomatology. In fact, people in their 50s who report low psychological well-being are more than twice as likely to suffer from depression when in their 60s, even after controlling for previous depression history, personality, demographic, economic, and physical health variables [12], suggesting that low well-being is a substantial risk factor for future depression. Depression is characterized by high levels of brooding, and often is associated with a ruminative thinking style, and attentional biases suggesting impaired attentional disengagement from negative information (see [13], [14] for review), which may contribute to the prolonged responses to negative emotional stimuli that have been observed both in psychophysiological and neuroimaging measures, such as prolonged pupil dilations and amygdala activation [15]–[18]. The link between low psychological well-being and the dysregulated emotion observed in depression is further supported by findings from the neuroimaging literature: those reporting higher levels of purpose in life show better regulation of the amygdala (a brain region involved in fear and anxiety-related processes) by the ventral anterior cingulate cortex, such that activity in the amygdala is reduced and the ventral anterior cingulate cortex is activated to a greater extent for negative relative to neutral pictures [19]. Moreover, high purpose in life was associated with slower judgments of the valence of negative relative to neutral pictures, suggesting that persons having goals and a sense of direction in life appraised the negative pictures as less salient and potentially less threatening than did persons with lower levels of purpose in life. Finally, whereas depressive symptomatology has been linked to decreased gray matter volume in the insula [20], purpose in life (as well as the other well-being dimensions of personal growth and positive relations with others) are positively associated with right insular gray matter volume [21].

How might purpose in life protect against depression, the body and brain ravages of growing older, and the accumulated toll of stress and challenges over the years? Based on the accumulating evidence, we hypothesize that one mechanism through which high purpose in life may protect against depression and the wear and tear of life stress is by providing a buffer from negative events, promoting reappraisal and motivated coping processes, decreasing brooding and ruminative thinking styles, supporting faster and better recovery, and thus increasing resiliency. Therefore, we hypothesize that higher levels of self-reported purpose in life will be associated with laboratory measures of emotional recovery, specifically, better automatic regulation of negative emotion as exhibited by better recovery from negative emotional stimuli. Importantly, this hypothesis combines phenomenologically-experienced aspects of well-being with objectively measured laboratory assessments of the time course of emotional responses, as this combination may offer unique windows on adaptive human functioning.

**Figure 1:**
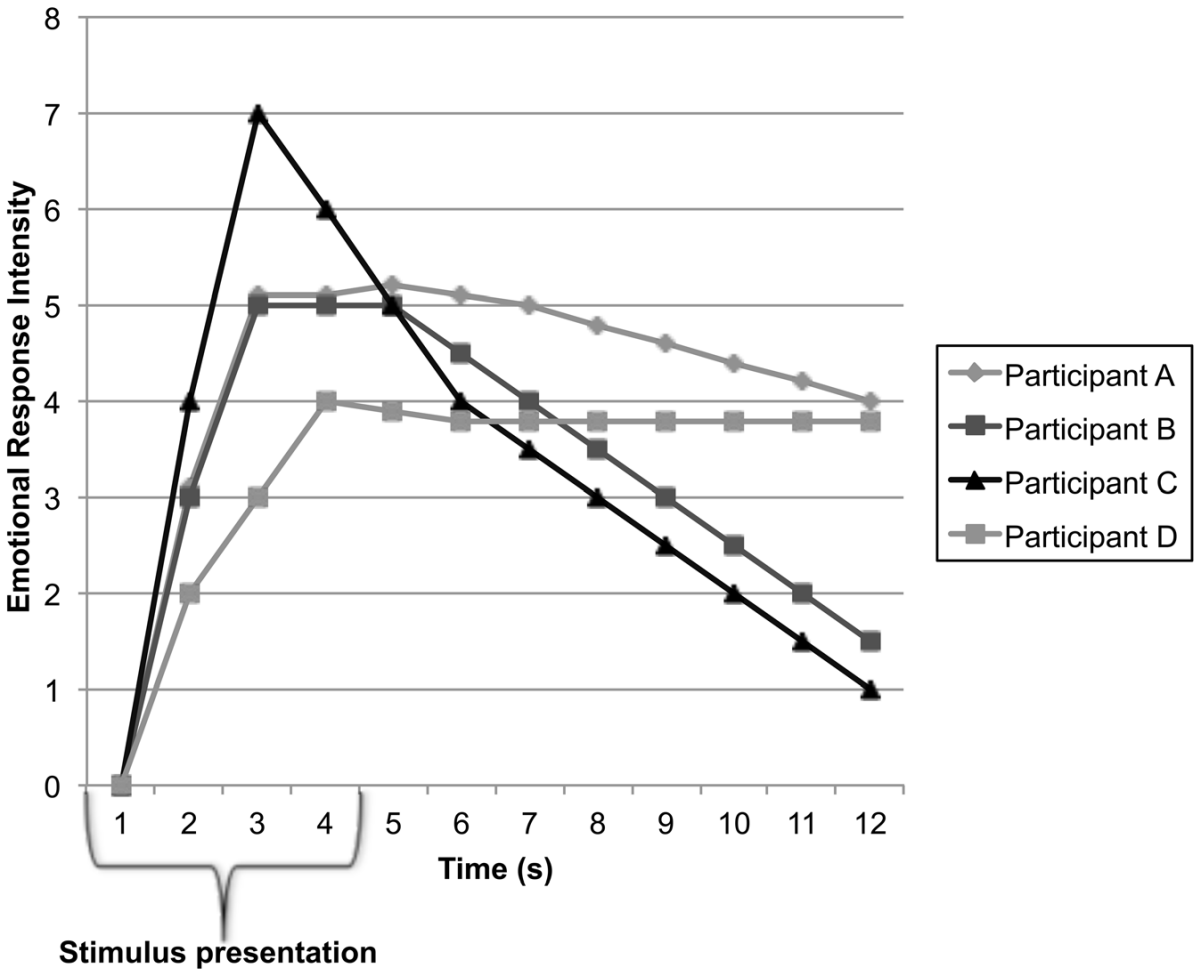
Hypothetical emotional response time courses from four different individuals to an emotionally provocative stimulus, such as a negatively-valenced picture displayed for 4 s as in the present study. Note that although subjects A and B have similar initial *reactivity* during the 4 s picture presentation period, after picture offset they differ in emotional *recovery.* Subject A shows a prolonged poor recovery, whereas Subject B recovers more rapidly. Subject C demonstrates greater initial reactivity with rapid recovery, whereas Subject D exemplifies an individual who may show smaller, blunted emotional reactivity but severely impaired recovery.

Heterogeneity is the rule in emotion research, characterized by large individual differences in how people react to the same emotional event or stimulus, and in how quickly and easily they recover from that stimulus (see Figure 1 for a hypothetical characterization of different emotional time course profiles to the same stimulus). While one person may briefly feel the effect of an unpleasant event, another may suffer a lingering and pervasive effect on mood. These individual differences in emotional reactivity and regulation constitute a person's affective style (see [22], [23] for theoretical reviews), may be critically influenced by a person’s sense of life purpose, and may also shape how much purpose and meaning one feels, suggesting bi-directional influences between these constructs

Affective psychophysiological research provides tools to measure an individual’s affective state without many of the demand characteristics biasing self-report (for review see [24], [25]), allowing for objective characterization of the time course of an individual’s emotional reactivity to and recovery from an emotion-eliciting stimulus [26] **.** Eyeblink reflex magnitude (EBR) measured to an acoustic startle probe from the *orbicularis oculi* muscle is emotion-modulated, such that activity is potentiated in the presence of an aversive stimulus and is diminished in the presence of a pleasant stimulus [27], [28]. Just as facial musculature recordings reflect a person’s affective state and their emotional response to stimuli, the temporal resolution possible with the EBR allows for differentiation of aspects of the emotional response from regulation of that response [26], [29], [30], providing objective estimates of both the magnitude and time course of emotional responses during and following incentives and challenges.

In the current paradigm, EBR measurements were obtained during the picture presentation period and after picture offset. We define emotional reactivity as reflected in measurements *during* the affective picture presentation when the emotionally evocative stimulus is present, and emotional recovery as measurements obtained *after* picture offset when the stimulus is no longer present. Parsing the time course in this way allows us to investigate individual differences in both reactivity and recovery. By including both the measures of reactivity and recovery in the same analytic models, we can examine individual differences in our measures during the recovery period unconfounded by variations in reactivity. Referring back to Figure 1, imagine two people who show similar reactions to the negative stimulus when it is present. One person’s regulatory capacities may facilitate quick recovery from a negative stimulus after it is removed (hypothetical subject B), while another may perseverate and show delayed recovery (hypothetical subject A), such as that observed in depression and dysphoria [15], [17], [31]. In this way, we can investigate the differential relationships between emotional reactivity and recovery with higher levels of purpose in life. We predicted that those subjects who reported higher levels of purpose in life would exhibit greater emotional recovery from the negative pictures, controlling for their initial reactivity to these pictures, thereby indicating a more adaptive emotion regulatory profile.

## Methods

### Ethics Statement

Ethical approval for telephone and mail surveys was obtained from the Social and Behavioral Science Institutional Review Board at the University of Wisconsin – Madison. All participants gave verbal consent, which included assurance of voluntary participation and confidentiality of data. The ethics committee approved the waiver of written consent. Such passive consent is customary for survey research by telephone and mail questionnaire. Ethical approval for the follow-up psychophysiological session was obtained from the Health Sciences Institutional Review Board at the University of Wisconsin – Madison and all participants provided written consent.

### Participants

The Survey of Midlife Development in the United States (MIDUS) began in 1995 with a national sample of Americans (*N* = 7,108) aged 25–74 years [32]. The majority (59.71%) was recruited through random digit dialing (RDD). The remaining respondents included siblings of the RDD sample and a large sample of twins (*N* = 1,914). Data collection focused on sociodemographic and psychosocial assessments obtained through phone interviews and self-administered questionnaires. In 2004, these survey assessments were repeated (MIDUS II). The retention rate from MIDUS I to MIDUS II was 75% (adjusted for mortality).

Psychophysiological data were collected on a subset of MIDUS II participants living in the Midwest who were able and willing to travel to our laboratory. The psychophysiology experiment followed the survey assessment on average over two years later (mean (SD) = 881 (26) days). A total of 331 (183 female) participants (age range 36–84 yrs, mean (SD) =  55.41 (11.12) yrs) agreed to participate in our experiment. For a variety of technical, responsivity, and other data quality issues, 253 (147 female/106 male; 185 singletons/68 twin or sibling) participants (age range 36–84 yrs, mean (SD)  = 54.68 (10.97) yrs) are included because they completed the psychological well-being questionnaire in the survey assessment and provided a total of 10 or more quantifiable eyeblink responses to the startle probes during the psychophysiological paradigm.

Data and documentation for MIDUS I and II, including all MIDUS projects, are publically available at the Inter-university Consortium for Political and Social Research (ICPSR; www.icpsr.umich.edu/icpsrweb/landing.jsp).

### Well-being

Given the growing focus on purpose in life as a key predictor of long-term health outcomes and underlying neurophysiology, our hypotheses targeted this particular dimension of well-being, although we included examination of all six scales of well-being collected in the survey assessments in MIDUS II (Scales of Psychological Well-Being; [3], [4]). Purpose in life refers to the tendency to derive meaning from life’s experiences and possess a sense of intentionality and goal directedness that guides behavior. The other five dimensions of psychological well-being included autonomy, environmental mastery, personal growth, positive relations with others, and self-acceptance. Each scale had seven items (internal consistency for these scales ranged from.69 to.85).

### Other Covariates

Other variables used in the analyses included age at the psychophysiological session, gender, the total number of valid eyeblink responses to the startle probes over the course of the psychophysiology experiment, the lag between the survey and psychophysiological assessments in days, trait positive and negative affect measured with the Positive and Negative Affect Schedule (PANAS; [33]), and subjective well-being measures including the Satisfaction with Life Scale [34] and an abbreviated version of the Gratitude Scale [35] asking participants to rate the following two statements: “I have so much in life to be thankful for” and “I am grateful to a wide variety of people.” The affect and subjective well-being measures were collected at the time of the psychophysiology session.

### Stimuli

A total of 90 International Affective Picture System pictures (IAPS; [36]) were presented in a randomized sequence. According to the IAPS normative ratings, 30 negative (mean (SD) = 2.89 (0.61)), 30 neutral (mean (SD) = 5.14 (0.52)) and 30 positive (mean (SD) = 7.24 (0.44)) pictures were selected, with the positive and negative pictures matched on arousal (negative pictures mean (SD) = 5.35 (0.54); neutral mean (SD) = 3.22 (0.73); positive mean (SD) = 5.23 (0.73)). All valences were matched on luminosity, complexity, and number of pictures with social content.

### Psychophysiological Procedure

**Figure 2:**
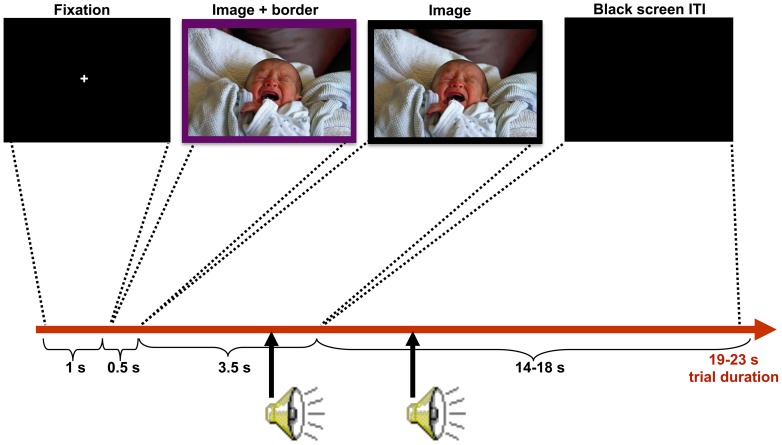
Psychophysiology paradigm. 30 positive, 30 negative, and 30 neutral pictures were displayed individually on separate trials. Participants responded as quickly as possible to the border color (purple or yellow) presented during the first 0.5 s of the picture presentation in order to maintain attention during the task. Startle probes were presented at 2900 ms after picture onset (assessing *reactivity*) and 1900 ms after picture offset (assessing *recovery*). Note: to avoid publication of an IAPS picture, the example negative picture was selected from the author’s personal collection to be representative of a prototypical IAPS picture. As the mother of the baby in the photograph, she has given written informed consent, as outlined in the PLOS consent form, to publication of their photograph.

The psychophysiological procedures have been described previously (see [30] for additional details). After informed consent was obtained, the participant completed questionnaires. The participant watched the positive, neutral, and negative pictures, and heard acoustic startle probes (50 ms, 105 dB, white noise bursts with very rapid onset time) presented through headphones. Each picture had either a yellow or purple border around it during the first 500 ms of the picture presentation, and participants responded as quickly as possible to the color of the border by pressing one of two keyboard buttons marked with the color with either their index or middle finger of their dominant hand. This color border identification task was used to keep subjects’ attention on the task and ensure they looked at the pictures. Pictures were presented on the screen for 4 s and were preceded by a 1 s fixation screen (see Figure 2 for a schematic of the psychophysiological paradigm’s design). Acoustic startle probes were inserted at three time points (randomized across trials to maintain an average inter-probe interval of ∼ 16 s). One probe occurred during the picture presentation (2900 ms following picture onset), a 2^nd^ probe occurred 400 ms after picture offset (4400 ms following picture onset), and a 3^rd^ probe occurred 1900 ms after picture offset (5900 ms following picture onset). A total of nine probes at each of the three time points were presented for each picture valence category, resulting in three non-probed trials for each picture valence. Because preliminary data analysis revealed reduced magnitude EBRs at the 2^nd^ probe (across all valences), these data were dropped from all further analyses because it suggests the 2^nd^ probe was affected by prepulse inhibition due to too close temporal proximity to the picture offset [37]. Participants who did not respond with a perceptible EBR on 10 or more of the 81-probed trials were excluded from EBR analyses as they were considered non-responders to the startle probe.

### Analytic Strategy

**Manipulation check.** We used a linear mixed-effects model to test the expected valence (negative, neutral, positive) modulation effect, a main effect of probe time (reactivity, recovery), and a valence x probe time interaction on EBR magnitude. The model included a family-specific random effect to account for within-family dependence between twins and siblings, as well as a participant-within-family-specific random effect to account for the within-person dependence between EBR measurements. Pairwise comparisons between valences (negative, neutral, and positive) and probe times (reactivity, recovery) were adjusted for multiple comparisons using the Bonferroni correction.

**Tests of purpose in life and the other psychological well-being dimensions predicting EBR measures of emotional reactivity and recovery to negative stimuli.** First, zero-order correlations were calculated between purpose in life and the other five psychological well-being dimensions with EBR magnitude measures obtained (1) at the reactivity probe, (2) at the recovery probe, and (3) with a recovery residual reflecting EBR magnitude at the recovery probe regressed on EBR magnitude at the reactivity probe to remove variation due to differences in reactivity (EBR magnitude at the reactivity probe and the recovery probe are inversely correlated, r = –0.15, p = 0.02).

Because age and gender have previously been shown to influence measures of emotional reactivity and recovery [29], [30], linear mixed-effects models were used to test the ability of each of the psychological well-being dimensions to predict EBR magnitude at the reactivity probe as well as EBR magnitude at the recovery probe on negative trials, while controlling for EBR magnitude at the reactivity probe (only included in the recovery models), age, gender, the total number of valid eyeblink responses to the startle probe over the course of the psychophysiology experiment, and the lag between the survey and psychophysiological assessments in days (A Models). The total number of valid eyeblinks was included as a covariate so that the results were not confounded with the reliability of the estimated EBR magnitude. Then to ascertain the specificity of purpose in life’s ability to predict recovery, we added the other five psychological well-being dimensions, both trait positive and negative affect (PANAS: mean positive affect and mean negative affect) and subjective well-being measures (Satisfaction with Life Scale mean and abbreviated Gratitude Scale mean) as covariates in one linear mixed-effects model (B Model) while including all of the covariates included in the earlier models. All models included a family-specific random effect to account for within-family dependence between twins and siblings. Finally, we used linear mixed effects models to control for data dependencies due to twins and siblings being included in the sample rather than randomly pick one person from each family cluster because the latter reduces the sample size, thereby compromising effect size estimates and power.

## Results

### Manipulation Check

**Figure 3:**
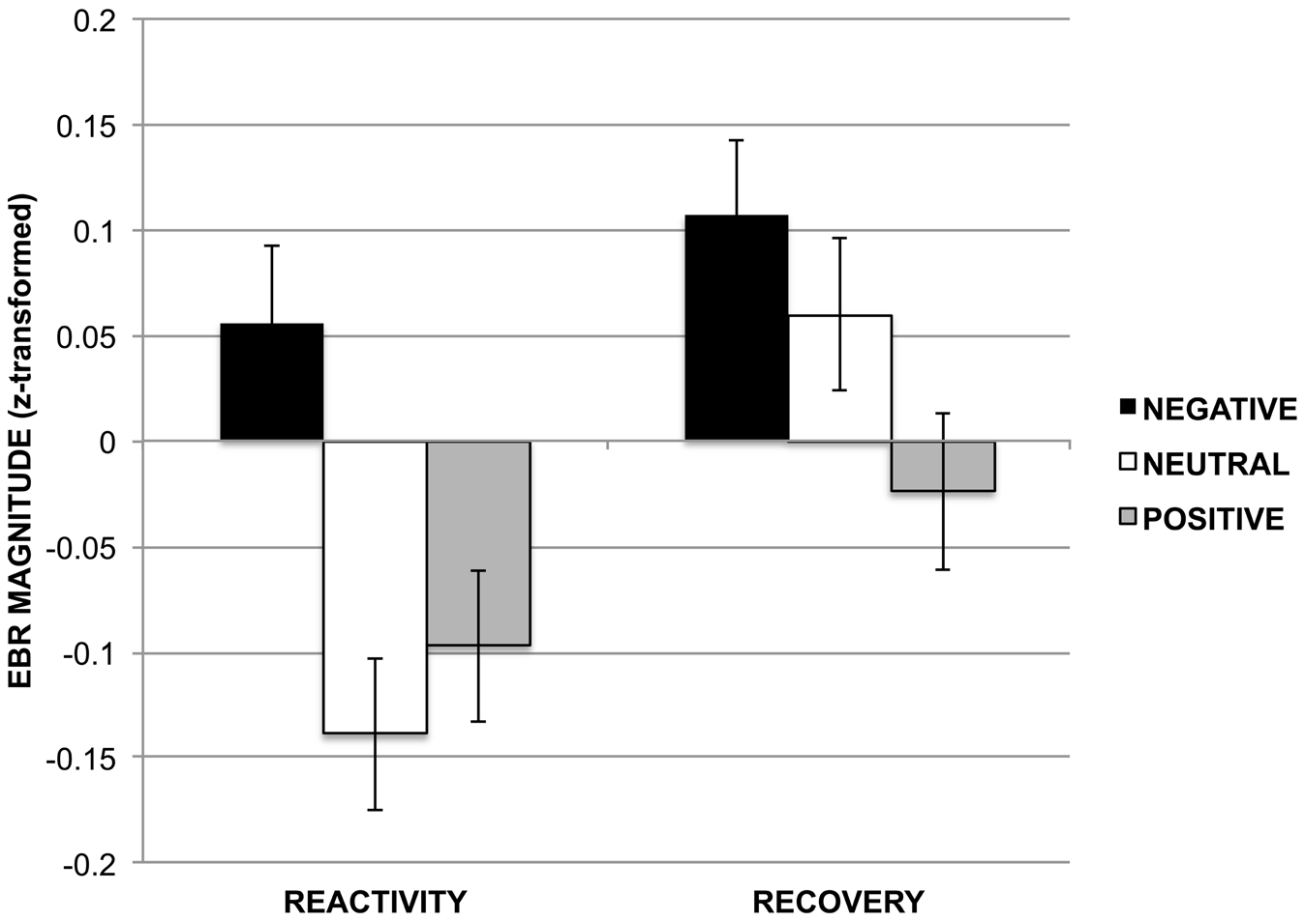
Manipulation check. Emotion modulation was observed in the EBR measures with a significant main effect of valence: EBR magnitude was greater on negative compared to both neutral and positive trials across probe times. A main effect of probe time showed larger EBR magnitude across valences in response to the recovery probe after picture offset when the computer screen was black than to the reactivity probe during the picture presentation (consistent with findings of larger responses to startling stimuli in darkness than in light). Mean EBR magnitude was significantly greater on negative than both neutral and positive trials at the reactivity probe, and greater on negative than positive trials at the recovery probe.

Emotion significantly modulated EBR magnitude. A significant main effect of valence was found across probe times, F(2,1527)  = 8.96, *p*<0.001, such that EBR magnitude was greater across probe times on negative (mean (SE)  = 0.08 (0.03)) compared to both neutral (mean (SE)  = –0.04 (0.03)) and positive (mean (SE)  = –0.06 (0.03)) trials. A significant main effect of probe time was found across valences, F(1,1527)  = 13.25, *p*<0.001, such that EBR magnitude was greater across valences during the recovery probe time after picture offset (mean (SE)  = 0.05 (0.02)) than the reactivity probe time during the picture presentation (mean (SE)  = –0.06 (0.02)), consistent with previous reports that startle responses are larger for humans in darkness than in light [38], reflecting the change in room light levels between a picture on the screen compared to a black computer screen. The valence x probe time interaction was marginally significant, F(2,1527)  = 2.42, *p* = 0.09. See Figure 3 for the EBR averages by valence and probe time.

### Effects of Purpose in life and the other Psychological Well-being Dimensions

**Figure 4:**
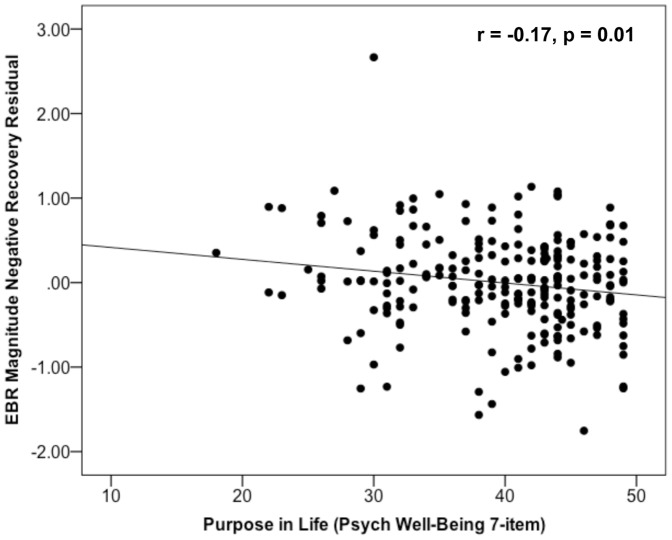
Purpose in life predicts smaller EBR indexing greater recovery after negative picture offset. Note: (1) EBR values are log-transformed and then z-scored within each participant. (2) The EBR recovery measure here reflects EBR magnitude at the recovery probe regressed on EBR magnitude at the reactivity probe, removing variation due to differences in reactivity. (3) The relation remains significant if the outlier is removed: Purpose in life x EBR negative magnitude residual at recovery r = –0.15, p = 0.03.

The zero order correlations between each of the psychological well-being dimensions with EBR magnitude measures obtained (1) at the reactivity probe, (2) at the recovery probe, and (3) with a recovery residual reflecting EBR magnitude at the recovery probe regressed on EBR magnitude at the reactivity probe to remove variation due to differences in reactivity are presented in Table 1. These correlations reveal that both purpose in life and positive relations with others predicted larger EBR magnitude during the picture presentations indicating greater reactivity to the negative pictures. Both purpose in life and self-acceptance also predicted significantly smaller EBR magnitude at the recovery probe, but only purpose in life predicted significantly smaller recovery residuals, when EBR magnitude at the recovery probe is regressed on EBR magnitude at the reactivity probe, controlling for differences in reactivity. See Figure 4 for a scatterplot of the linear relations between purpose in life and the EBR recovery residual.

**Table 1:** Zero order correlations between the individual dimensions of psychological well-being and EBR magnitude measures of emotional reactivity and recovery on negative picture trials.

Dimension of Well-Being	EBR Magnitude @ Reactivity	EBR Magnitude @ Recovery	EBR Recovery Residual*
***Purpose in Life***	**0.13 (.04)**	–**0.19 (.003)**	–**0.17 (.01)**
Autonomy	0.03 (.70)	–0.03 (.62)	–0.04 (.55)
Environmental Mastery	0.04 (.52)	–0.07 (.28)	–0.08 (.21)
Personal Growth	0.07 (.26)	–0.10 (.11)	–0.11 (.10)
Positive Relations with Others	**0.12 (.05)**	–0.09 (.15)	–0.08 (.23)
Self-Acceptance	0.09 (.18)	–**0.12 (.05)**	–0.10 (.12)

Note: Correlations with the r (p) values in bold indicate p<0.05.

* The EBR recovery measure here reflects EBR magnitude at the recovery probe regressed on EBR magnitude at the reactivity probe, removing variation due to differences in reactivity. (EBR magnitude at the reactivity probe and the recovery probe are inversely correlated, r = –0.15, p = 0.02).

When demographic and data quality variables were included as covariates, the linear mixed-effects models testing the ability of each of the well-being dimensions to predict reactivity were not significant (all **β**s<0.009, all *p*s > 0.13). However, the estimates presented in Table 2 show that when demographic, data quality, and EBR magnitude at the reactivity probe were included as covariates in the models with EBR magnitude at the recovery probe, purpose in life still predicted smaller EBR at the recovery probe; A Models: **β** = –0.016, *t*(230)  = –3.02, *p* = 0.003. Personal growth and self-acceptance also predicted EBR magnitude at the recovery probe in the A Models, however only purpose in life remained at trend level when all of the above covariates were included as well as all of the psychological well-being dimensions, trait positive and negative affect, and subjective well-being variables in the same model, **β** = –0.017, *t*(216.90)  = –1.86, *p* = 0.064 (see Table 2). Thus, the findings from the linear mixed-effects model, which adjusts for the potential dependencies in the data from twins and siblings, show that when all of the psychological well-being dimensions, subjective well-being, and trait positive and negative affect are included in the same analytic model, purpose in life still predicts at trend level lower EBR magnitude at recovery. Therefore, higher levels of purpose in life are associated with better recovery from negative emotional stimuli even with the effect of the other well-being dimensions and positive and negative trait affect removed.

**Table 2:** Estimates (p Values) for each of the individual dimensions of psychological well-being from linear mixed-effects models predicting EBR magnitude measures of recovery from negative pictures.

Dimension of Well-Being	*A Models:* β (p)*	*B Model*: β (p)**
***Purpose in Life***	–**0.016 (.003)**	–0.017 (.06)†
Autonomy	–0.004 (.46)	0.005 (.42)
Environmental Mastery	–0.006 (.19)	0.003 (.71)
Personal Growth	–**0.012 (.02)**	–0.004 (.61)
Positive Relations with Others	–0.009 (.08)	0.003 (.76)
Self-Acceptance	–**0.009 (.04)**	–0.007 (.43)

Note: **β** (p) values in bold indicate p<0.05.

* *A Models* include covariates controlling for the EBR magnitude at the reactivity probe, age, gender, the total number of valid eyeblink responses to the startle probe over the course of the psychophysiology paradigm, and lagtime in days between the survey and psychophysiological assessments in a separate model for each of the psychological well-being dimensions predicting EBR magnitude at the recovery probe.

** *B Model* includes all of the covariates included in the A models, as well all 6 of the psychological well-being dimensions, trait positive and negative affect, and subjective well-being variables in the same model predicting EBR magnitude at the recovery probe.

† If the one outlier participant on EBR magnitude at recovery is removed from this model, purpose in life significantly predicts EBR magnitude at recovery: **β** = –0.017, *t*(216.61)  = –2.05, *p* = 0.04. Even with the outlier removed, none of the other well-being measures significantly predicted EBR recovery.

Finally, as seen in Figure 4, there was one outlier on EBR magnitude at the recovery probe. All of the analyses reported above include the outlier. When the outlier participant’s data is excluded from the linear mixed-effects models, purpose in life still significantly predicted EBR recovery in each of the models: A Model linear mixed-effects model: **β** = **–**0.014, *t*(229) = –2.64, *p* = 0.009; and B Model linear mixed-effects model: **β** = **–**0.017, *t*(216.61)  = –2.05, *p* = .042. Importantly, only purpose in life and none of the other well-being dimensions predicted EBR recovery in the B Model when all of the well-being dimensions were included in the same model, regardless of whether the outlier was included or not, all *p*s > 0.43.

## Discussion

Higher levels of purpose in life, self-reported on average over two years prior, predicted better recovery from a negative stimulus measured with the eyeblink startle response (EBR), such that those persons reporting greater life purpose exhibited smaller EBR magnitude after picture offset. The significant association between purpose in life and EBR magnitude measures of recovery were observed even when differences in EBR magnitude at the reactivity probe during the stimulus presentation were statistically removed. Moreover, the relations between purpose in life and emotional recovery from negative stimuli were still significant when controlling for participants’ age, gender, the five other psychological well-being dimensions, self-reported trait positive and negative affect, and measures of their subjective well-being.

The better recovery exhibited after stimulus offset by those with high life purpose reflects a more healthy emotional time course profile. Davis has demonstrated a crucial role of the central nucleus of the amygdala in the fear potentiation of the startle response in rats [39], and human studies suggest a similar role for the amygdala in emotion-modulated startle [40]–[42]. Thus the current finding of reduced startle during recovery is consistent with previous reports from our laboratory [19] showing better regulation of the amygdalar response to negative pictures in people with high levels of purpose in life.

Purpose in life stood out among the well-being measures in its ability to predict EBR measures of recovery, suggesting that feeling purpose and meaning in one’s life may contribute to a more healthful and adaptive regulation of negative emotional responses. Taubitz, Robinson, and Larson (2013) recently examined the time course of EBR emotion-modulation in dysphoric women by examining both reactivity to the picture presentation and recovery after picture offset [33]. Dysphoric females, compared to non-dysphoric females, exhibited blunted EBR for negative relative to neutral pictures during the picture presentation (less reactivity), but heightened EBR for negative relative to neutral pictures after picture offset (poorer recovery), as demonstrated by hypothetical Participant D in Figure 1. In other words, dysphoric females exhibited a recovery profile comparable to that which persons with low purpose in life exhibited in the current study. However, it is important to point out that in the current study, the significant relations between purpose in life and recovery were observed even when gender and both trait positive and negative affect were controlled.

Poor recovery from negative stimuli has been observed in depressed individuals who display sustained pupil dilation and amygdala activation to negative words [15]–[17]. While nondepressed individuals increase dorsolateral prefrontal cortex activation and decrease amygdala activation when reappraising emotional pictures, depressed persons do not [18]. Depression is also characterized by working memory and attentional biases, including increased elaboration of negative information, problems disengaging from negative material, and deficits in cognitive control when processing negative information [13], [14]. Individuals with depression usually report high levels of rumination [43] and greater use of emotional suppression [44]. Both rumination and suppression are ineffective emotion regulation techniques that can actually increase negative emotions [45] and the associated sympathetic nervous system activity [46], [47], suggesting a potential mechanism underlying the physical burden of the dysregulated negative affect characterizing the disorder. According to the World Health Survey, depression has a greater impact on overall health than arthritis, diabetes, angina, and asthma [48]. Because lower levels of psychological well-being, including lower levels of purpose in life, are correlated with higher rates of depression [3], [4], [11], [12], [49], the psychological well-being and depression literature further supports the linkage between emotional regulatory skills and purpose in life.

How might higher levels of purpose in life contribute to the ability to recover from aversive and unpleasant events? Additional research is needed. However, having greater purpose in life may provide motivation to constructively learn from and reappraise negative events in an adaptive manner and avoid brooding and ruminative tendencies, so as to quickly refocus on one’s goals and purpose. Possessing higher levels of well-being, especially purpose in life, may provide a wealth of resources one can use to cope with the current situation, motivating an adaptive and proactive handling of the situation, buffering the effect of adverse experiences, and thereby facilitating and fostering the learning and development of even greater emotion regulation skill over time. This idea is supported by reports that purpose in life is a key factor associated with better recovery from trauma in at-risk African American populations [50] as well as Pakistani earthquake survivors [51], such that those reporting higher levels of purpose in life had decreased rates of post-traumatic stress disorder (PTSD) after suffering trauma. Purpose in life might be a “resilience factor,” protecting against the development of psychopathologies such as PTSD and depression after stress and trauma exposure, or even the repeated minor stresses experienced over the course of a lifetime. The current study suggests a mechanism through which purpose in life may confer protection is by facilitating automatic emotion regulation after negative emotional provocation.

In turn, the reverse may also be true, to experience high well-being and purpose, individuals may need to be able to flexibly modify their emotional responses depending on the situation [52] whether it is to (i) temporarily up-regulate and increase a negative emotion to empathize with another or (ii) quickly down-regulate and decrease a negative emotion to refocus attention and concentrate on a task after experiencing an unpleasant event. Thus, people more skilled and adept at emotion regulation may have advantages in work and family life that nurture greater life success, including a greater sense of mastery, growth, and especially purpose in life. Moreover, the connections between purpose in life and emotional recovery/regulation are likely reciprocal in nature over time. Those with a greater sense of purpose in life may be better prepared to respond to emotional challenges more quickly and efficiently as our data show. However, better emotional recovery from negative stimuli, particularly cumulatively through development, might also lead to greater purpose in life. Our study tested the ability of purpose in life, measured about two years prior, to predict emotional recovery, underscoring the need for additional studies to test whether the relationship between purpose in life and emotion regulatory abilities are uni- or bidirectional, especially at different stages in the life span.

In conclusion, this longitudinal investigation combined phenomenologically-experienced aspects of well-being with automatic, objectively measured assessments of emotional reactivity and recovery obtained in the laboratory over two years after the well-being assessment. The MIDUS study features a remarkably large sample with a wide age range, unique for an experiment utilizing psychophysiological measures. Our findings suggest that higher levels of self-reported purpose in life predict a person’s future ability to recover from exposure to negative stimuli. Persons with higher purpose in life showed a facilitated recovery with smaller eyeblink startle responses after negative stimuli offset, suggesting a healthier overall emotional time course. Additional research testing potential mechanisms by which purpose in life may be related to emotional recovery skills [12] and confer resilience from trauma [50], [51], as well as how better emotional recovery skills may contribute to purpose in life is warranted. Purposeful life engagement has increasingly been linked to better health outcomes, including assessments of morbidity and mortality [7], [10] as well as intervening biological mechanisms [9], [53]. Understanding brain-based emotion regulation processes that contribute to these outcomes are important next steps.
